# Molecular Characteristics of Methicillin-Resistant *Staphylococcus epidermidis* on the Abdominal Skin of Females before Laparotomy

**DOI:** 10.3390/ijms17060992

**Published:** 2016-06-22

**Authors:** Pin-Jia Wang, Cheng-Bin Xie, Feng-Hui Sun, Li-Juan Guo, Min Dai, Xi Cheng, Yong-Xin Ma

**Affiliations:** 1 Medical Genetics, West China Shool of Medicine, West China Hospital, Sichuan University, Chengdu 610041, China; pinjiawong@foxmail.com; 2 School of Medical Laboratory Science, Chengdu Medical College, Chengdu 610500, China; sunfenghui_83@hotmail.com (F.-H.S.); gljemily@163.com (L.-J.G.); daimin1015@163.com (M.D.); cissi7@foxmail.com (X.C.); 3 Department of Laboratory Medicine, Sichuan Provincial Hospital for Women and Children, Chengdu 610045, China; chengbinxie@foxmail.com

**Keywords:** methicillin-resistant *Staphylococcus epidermidis*, SCC*mec*, PFGE, skin, laparotomy, antibiotic resistant

## Abstract

*Staphylococcus epidermidis*, especially methicillin-resistant strains, may be the source of surgical site infections and may be a reservoir of staphylococcal cassette chromosome *mec* (SCC*mec*) for *S. aureus*. The aim of this study was to investigate the prevalence of methicillin-resistant *S. epidermidis* (MRSE) on the abdominal skin of females before laparotomy and determine the molecular characteristics and antimicrobial susceptibility patterns of these isolates. MRSE was found in 54 of 157 isolates based on *mecA* gene detection, and there was no difference in *icaA* gene carriage rate between MRSE and methicillin-susceptible *S. epidermidis* (MSSE) isolates. Antimicrobial susceptibility profiles were determined by broth microdilution antimicrobial susceptibility testing according to the latest CLSI manuals. All MRSE isolates had unfavorable antimicrobial susceptibility patterns. Twenty-three MRSE strains (42.6%) were multi-drug resistant. SCC*mec* typing and pulsed field gel electrophoresis (PFGE) typing was performed. Thirty-nine (72.2%) had a single SCC*mec* type, whereas 1.9% had two types. Fourteen strains (25.9%) were non-typeable (NT). The most frequent MRSE genotype was SCC*mec* type IVa. High diversity with PFGE patterns was obtained for MRSE, and there were no isolates exhibiting identical pulsotype. The results confirm that methicillin-resistant strains are frequently present among *S. epidermidis* on the abdominal skin of females before laparotomy. Moreover, resistance profiles seem to have no association with the SCC*mec* types or PFGE types for most common antibiotics.

## 1. Introduction

Surgical interventions are common in the day-to-day practice of obstetrics and gynecology, and surgical site infections (SSIs) are frequent complications of abdominal gynecological operations. Surveys of postoperative wound infections in obstetrics and gynecology departments showed that the overall wound infection rate was 5.87% to 12.6% [1,2,3]. Postoperative wound infections can increase the costs and hospital stay. Moreover, they lead to higher morbidity and lower life quality of surgical patients. The widespread use of antibiotic prophylaxis has reduced but not eradicated the prevalence of serious postoperative infections.

For most gynecological SSIs, the source of pathogens is the endogenous flora of the patient’s skin or vagina [4]. When the skin or vagina is incised, the exposed tissues are at risk for contamination with endogenous flora. *S. epidermidis* is the most prevalent and persistent species found on the human skin and mucous membranes, constituting 65% to 90% of all staphylococci isolated from these environments [5], and it may act as a source of later bacteremia and surgical site infections. The majority of cases reported identified *S. epidermidis* as a common bacterium responsible for wound infections after surgery [6,7,8]. In one study by Martens *et al.* [9], 64% infected wounds after cesarean delivery had positive bacterial cultures, with *S. epidermidis* (29%) being the most frequent isolates.

In recent decades, *S. epidermidis* has emerged as the cause of hospital-acquired infections. Virulence is mainly attributed to biofilm formation based on polysaccharide intercellular adhesin (PIA), which is encoded by *S. epidermidis icaADBC* operon genes. About 75%–90% of *S. epidermidis* strains circulating in hospitals have been estimated to be methicillin-resistant [10,11]. The methicillin-resistance to *S. epidermidis* is usually due to the *mecA* gene, which is carried by staphylococcal cassette chromosome *mec* (SCC*mec*) and produces a special penicillin binding protein 2a (PBP2a) with low affinity for β-lactams. SCC*mec* can also carry genetic elements for other antibiotic resistance; therefore, most of the methicillin-resistant strains (MRSE) are also highly resistant to other antibiotics. In diverse collections of *S. epidermidis* isolates, molecular characterization of this element revealed five SCC*mec* types previously found in *S. aureus* [12]. Additionally, there were high numbers of new and non-typeable SCC*mec* types [13,14,15,16]. These results indicate a high degree of genetic diversity within the SCC*mec* elements carried by *S. epidermidis*. Therefore, it is imperative that the genetic diversity of MRSE isolates should be determined by using a gold standard genotyping technique, such as pulsed field gel electrophoresis (PFGE).

Several studies have described the antimicrobial susceptibility of bacteria isolated from SSIs and prevalence of staphylococcal strains in the surgical site, but antimicrobial susceptibility and molecular characteristics of MRSE on the abdominal skin of females before laparotomy are extremely limited, which fails to guide infection control for gynecological SSIs. Therefore, the aim of this study was to investigate the prevalence of MRSE on the abdominal skin of females before laparotomy and determine the molecular characteristics and antimicrobial resistance patterns of these isolates.

## 2. Results

### 2.1. SCCmec Typing of MRSE

A total of 157 *S. epidermidis* isolates were found on the abdominal skin of 160 females (98.1%). Fifty-four isolates were positive for *mecA* and were thus classified as MRSE (34.4%), 13 isolates were positive for *icaA* (8.28%), and no difference was observed in *icaA* positive rates between MRSE and methicillin-susceptible *S. epidermidis* (MSSE) isolates (respectively 5 and 8, *p* > 0.05). Among MRSE isolates, established SCC*mec* types were detected in 40 isolates (74.1%), with 1.9% cassettes of type I, 1.9% cassettes of type II + IVb, 7.4% cassettes of type III, 51.9% cassettes of type IVa, 5.6% cassettes of type IVd, and 5.6% cassettes of type V. The remaining 14 isolates (25.9%) carried non-typeable (NT) cassettes. SCC*mec* type IV (including IVa and IVd) (*n* = 31) was predominant among the MRSE isolates from abdominal skin of females, followed by NT (*n* = 14), III (*n* = 4), V (*n* = 3), and I and II+IVb (*n* = 1 each).

### 2.2. Antimicrobial Susceptibility of MRSE

All of the 54 MRSE isolates were susceptible to some non-β-lactam antibiotics, such as rifampicin, gatifloxacin, moxifloxacin, quinupristin–dalfopristin, linezolid, and vancomycin. The majority of the MRSE isolates were resistant to many common antibiotics, such as penicillin, ampicillin, erythromycin, azithromycin, and trimethoprim–sulfamethoxazole. Resistance rates of the MSSE isolates were significantly less than those of the MRSE isolates to ciprofloxacin, levofloxacin, gentamicin, erythromycin, azithromycin, and trimethoprim–sulfamethoxazole (*p* < 0.05). The results indicated that most of the non-β-lactam antibiotic resistances were correlated with the *mecA* gene, just as β-lactam antibiotics. Resistance rates for the remaining antibiotics in the MSSE and MRSE isolates are shown in Table 1. We found that the susceptibility to oxacillin was variable in MRSE with minimal inhibitory concentrations (MICs) ranging from 0.25 to 4 μg/mL. Carriages of *mecA* in these isolates were not always associated with significant resistance to oxacillin. Five of the *mecA* positive *S. epidermidis* showed oxacillin sensitivity.

*S. epidermidis* carrying SCC*mec* type I, type II + IVb, type III, type V, and type NT cassettes were all resistant to penicillin and ampicillin, whereas each of type IVa and type IVd was susceptible. Except for those β-lactam antibiotics, SCC*mec* type IVa and type NT MRSE isolates had almost identical resistance rates to gentamicin, ciprofloxacin, levofloxacin, azithromycin, erythromycin, chloramphenicol, tetracycline, and trimethoprim–sulfamethoxazole (*p* > 0.05). The results indicate that resistance profiles seem to have no association with the SCC*mec* types for most common antibiotics. However, more notably, the resistance rate to clindamycin of the SCC*mec* type IVa was significantly lower than that of the SCC*mec* type NT MRSE isolates (*p* < 0.01). The details of the resistance rates among MRSE with different SCC*mec* types are summarized in Table 2.

Thirty different resistance patterns were obtained for all the MRSE isolates, and twenty-three isolates (42.6%) were multi-drug resistant. However, only 17 resistance patterns for the MSSE isolates and 10 isolates (9.7%) were multi-drug resistant. The resistance patterns were quite different in between, but detailed data are not shown here. SCC*mec* types and PFGE types associated with these resistance patterns are shown in Table 3. There are seven isolates only resistant to oxacillin, erythromycin, and trimethoprim–sulfamethoxazole. Patterns with ERY resistance, OXA + CLI resistance, OXA + SXT resistance, OXA + SXT + ERY resistance, and OXA + GEN + CIP + SXT + ERY resistance appear more frequently in SCC*mec* type IVa isolates. Resistance patterns seem to have no relationship with the PFGE types.

### 2.3. PFGE of MRSE

**Figure 1:**
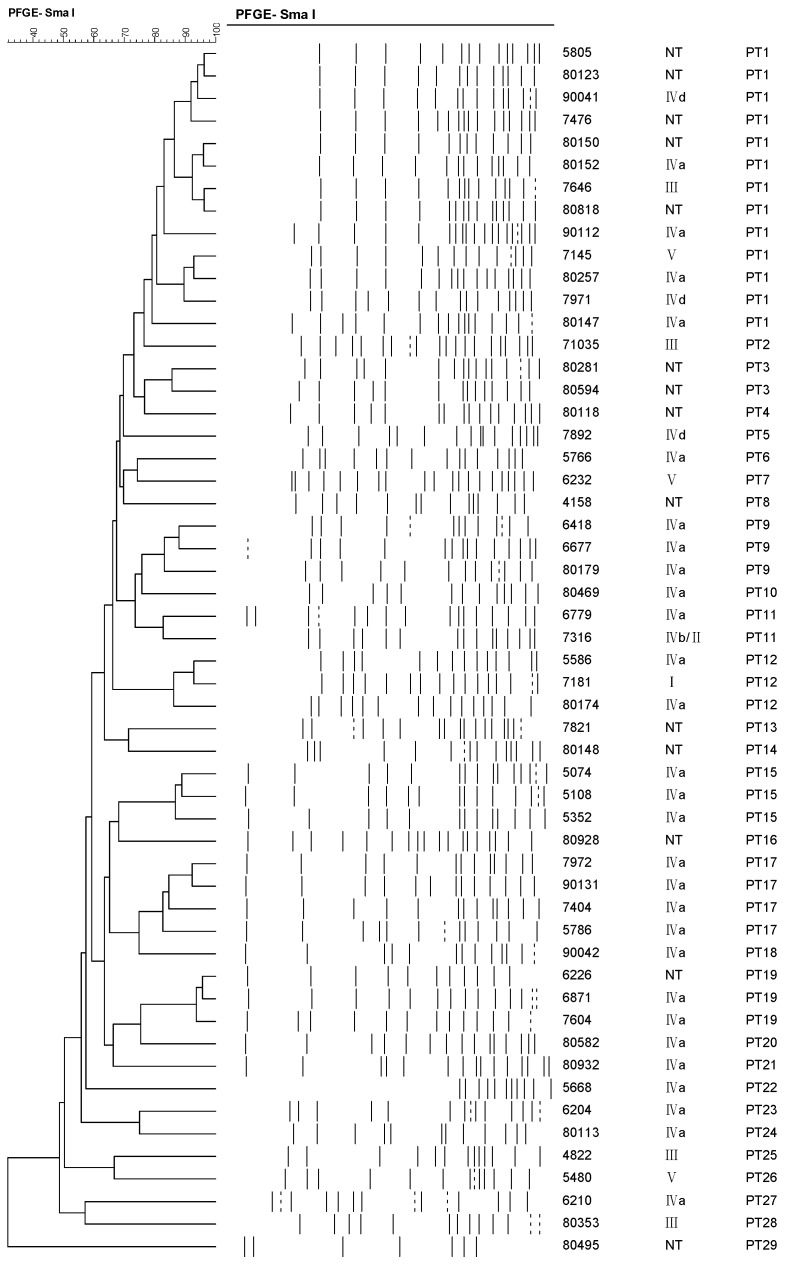
Dendrogram of pulsed-field gel electrophoresis patterns of MRSE isolates. At a Dice similarity index of 79%, 29 PFGE types were identified. The strain number, the SCC*mec* type, and the PFGE type are indicated at the right of the dendrogram.

PFGE for MRSE was performed and analyzed with a Dice similarity index of 79%, and 54 MRSE isolates were clustered in 29 PFGE types (PT). There were no isolates with identical pulsotype, and the results showed a notable genetic diversity among the isolates (Figure 1). There were 21 PFGE types consisting of only a single *Sma*I pattern. The remaining PFGE types groups consisted of 2 to 13 *Sma*I-PFGE patterns. The most frequent PFGE types were pulsotype PT1 (*n* = 13), followed by types PT17 (*n* = 4), 9, 12, 15, and 19 (*n* = 3 respectively). Except for PT1, 11, 12, and 19 isolates, most of the PFGE types consisted of one SCC*mec* cassettes. A relatively high similarity of pulsotype was found in the predominant SCC*mec* type IVa. Four isolates with SCC*mec* IVa were grouped in PT1 with a similarity of 79.09%, and four isolates were grouped in PT17 with similarity of 82.67%. Three isolates with SCC*mec* IVa were grouped in PT9 with a similarity of 83.22%, and three isolates were grouped in PT15 with a similarity of 86.76%. Two isolates with SCC*mec* IVa were grouped in PT12 with a similarity of 86.21%, and two were grouped in PT19 with a similarity of 93.66%.

## 3. Discussion

The human body is the habitat of large and varied populations of bacteria that are at the same time both potentially dangerous and helpful to human health. In recent years, there has been a strong interest to fully characterize the bacteria strains associated with different parts of the body under different health conditions [17,18,19]. *S. epidermidis* is the most significant member of the coagulase negative staphylococci (CoNS) and part of normal skin flora, and it has a benign relationship with the host, but it has emerged as the cause of serious infections, such as surgical wound infections. From our previous investigation, *S. epidermidis* was frequently isolated from peritoneal fluid of females with pelvic inflammatory disease, such as abdominal pain, fever, and foul-smelling vaginal discharge. Our observations also matched those of Szumała’s investigation; they found that *S. epidermidis* was one of the most common bacteria (34.1%) [20]. Therefore, we believe that *S. epidermidis* might be significantly associated with pelvic inflammatory disease or postoperative intra-abdominal infections of females.

This prospective study included 160 females before laparotomy due to ectopic pregnancy, caesarean section, uterine tumors, or pelvic inflammatory disease, to which little attention had been paid in previous reports. In this study, the carriage rate of *S. epidermidis* on the abdominal skin of these females was as high as 98.1% (157/160), the positive rate for *mecA* of *S. epidermidis* was as high as 34.4% (54/157); however, the positive rate for *icaA* was as low as 8.28% (13/157). However, it is worth noting that there was no difference in *icaA* positive rates between MRSE and MSSE isolates (respectively 5 and 8, *p* > 0.05), which means that the *icaA* gene could not be associated with *mecA* gene for these isolates. Some previous reports have identified that horizontal transfer of SCC*mec* elements occurs between *S. aureus* and methicillin-resistant coagulase-negative *Staphylococci* [16,21]. SCC*mec* elements are very common in *S. epidermidis* in this study, which may act as an available reservoir for *S. aureus* to develop methicillin resistance [22,23]. Thus, it is very important for medical staff to understand the resistance profiles and genotype of MRSE of females before laparotomy to guide infection control and the reasonable use of antibiotics.

The circulation of different SCC*mec* types of MRSE varies in the population, environment, and geographical locations. SCC*mec* types have been used to distinguish community-associated methicillin resistant *Staphylococci* (CA-MRS) from healthcare-associated methicillin-resistant *Staphylococci* (HA-MRS). MRSA bacteremia with SCC*mec* II/III isolates has occurred more among patients with serious comorbidities and prolonged hospitalization, while community onset, skin and soft tissue infection, and deep-seated infection best predicted SCC*mec* IV/V MRSA bacteremia [24]. SCC*mec* type IV was common in MR-CoNS. Data from Jamaluddin [25] confirmed that SCC*mec* IVa was found in most community-acquired MRSE. Other researchers reported that 36% of MRSE harbored SCC*mec* type IV, and type IV was common among a geographically dispersed collection of *S. epidermidis* isolates [26]. Epidemiological studies and molecular characterization of methicillin-resistant staphylococci from healthy Jordanian population showed the MR-CoNS carriage was 54.2% and these isolates were characterized by single, double, and un-typeable SCC*mec* elements, with *S. epidermidis* SCC*mec* type IVa predominating [27]. In agreement with previous reports, this study found that SCC*mec* types I and II were few, while type IV was relatively common. SCC*mec* type IVa was predominant among MRSE isolates from the abdominal skin of females. A report from Shitrit [28] confirmed that patients with SCC*mec* type IV had fewer previous hospitalizations, less antibiotic treatment, and fewer invasive procedures, as well as fewer comorbid conditions and a shorter length of stay compared with other SCC*mec*-type patients.

In this study, SCC*mec* types were determined for most of the isolates (40 of 54), and the remaining isolates (25.9%) could not be amplified by this M-PCR. This rate was consistent with a previous report with the same M-PCR [28]. Several previous studies have demonstrated that there is a higher frequency of non-typeable and new SCC*mec* types among *S. epidermidis* or other coagulase-negative staphylococci. Therefore, a considerable proportion of MRSE isolates cannot be assigned by the currently available PCR-based methods [29], which means to us that a new SCC*mec* typing technique especially for *S. epidermidis* should be developed as soon as possible. In this study, there was one isolate with two SCC*mec* type II+IVb, and it is likely that the two SCC*mec* elements actually constitute a composite rather than two independent units [29].

Pulsed-field gel electrophoresis has been considered the gold standard for the molecular typing of *S. epidermidis* for many years. Here, using a high-resolution PFGE method, a high degree of genetic diversity of MRSE isolates was found. We observed 29 pulsotypes for these MRSE isolates, and there were no isolates with an identical pulsotype, which showed a high diversity in genotype of MRSE isolates. The diversity in this study indicated that there were no immediate transmissions of any MRSE isolates among females in the same hospital and that the research data were relatively valid, reliable, and representative.

The MRSE isolates in this study had an unfavorable antimicrobial susceptibility patterns. Although they were completely sensitive to some non-β-lactam antibiotics, such as rifampicin, gatifloxacin, moxifloxacin, quinupristin–dalfopristin, linezolid, and vancomycin, more than 50% of SCC*mec* IVa MRSE strains were resistant to many common antibiotics in clinic, such as penicillin, ampicillin, oxacillin, trimethoprim–sulfamethoxazole, azithromycin, and erythromycin. Moreover, the isolates with SCC*mec* type IV (including IVa and IVb) in this study, which are usually identified as community-acquired strains, were resistant to many non-β-lactam antibiotics. By contrast, previous studies showed that SCC*mec* types IV are generally susceptible to non-β-lactam antibiotics [30]. The research data suggested that SCC*mec* type IV strains should acquire resistance to non-β-lactam antibiotics in order to accommodate hospital environments and excessive antibiotic exposure. Comparing SCC*mec* type IVa with SCC*mec* type NT isolates, we found that they had almost identical resistance rates to many non-β-lactam antibiotics, which suggested that resistance profiles seem to have no association with the SCC*mec* types for most of the common antibiotics for these isolates. It could also be, of course, that the numbers of different SCC*mec* type isolate are not big enough to show the difference between resistance profiles and SCC*mec* types. However, more notably, the resistance rate to clindamycin of SCC*mec* type IVa was significantly less than that of SCC*mec* type NT MRSE isolates.

As is known to all, inappropriate and broad-spectrum antibiotic therapy may not only disrupt the normal balance of bacteria of the human body, but also facilitate colonization by multi-drug resistant bacteria. The patients who were receiving an appropriate antibiotic treatment could not be colonized by MRS strains until they have been exposed to these strains, so MRSE strains may seriously threaten the patients, especially those who will receive surgery. The research data in this study suggested that all preoperative patients including pregnant females may be the potential and important reservoirs of MRSE in hospital and community. It is important for medical staff to select appropriate antibiotic prophylaxis and conduct effective disinfection for preoperative patients to prevent and control the infection and spread of MRSE.

Consequently, although this study was performed at a single-center level and only concentrated on preoperative female patients, which means that data collection may be restricted and incomplete for some reasons, and different conclusions may be reached in a multivariate analysis if we expanded the number of enrolled cases, the research data in this study was able to clearly reflect the resistant patterns and genotype of MRSE of females before laparotomy. This study can provide medical staff with a better understanding of molecular epidemiology of MRSE in this region, which is very essential and meaningful for them to detect, treat, control, and prevent surgical site infections caused by this organism timely and efficiently.

## 4. Materials and Methods

### 4.1. Specimen Collection

This was a prospective cross-sectional study involving abdominal skin specimen of females from the age of 19 to 58 without skin or soft tissue infections and who were at the obstetrical or gynecological department from January to September 2015 in Sichuan Provincial Hospital for Women and Children, Chengdu, Sichuan, China. Abdominal surgeries were performed on these females due to ectopic pregnancy, caesarean section, uterine tumors, or pelvic inflammatory disease. The females provided written informed consent to participating in this study. Tegumentary specimens were obtained from abdominal incisions or puncture sites with moistened (sterile saline) swabs, which were directly inoculated onto Columbia blood agar.

### 4.2. Bacterial Isolation

*S. epidermidis* were identified by conventional methods after incubation for 24~48 h at 35 °C. Preliminary screening was based on Gram staining, catalase, plasma coagulase, and glucolysis tests. Final confirmation of *S. epidermidis* was performed with the API 20 Staph kit (Biomerieux, Marcy-l'Étoile, France). A total of 157 *S. epidermidis* non-duplicate isolates were recovered from the abdominal skin in females before laparotomy. All strains were stored in glycerin-buffered saline at −70 °C and were subcultured twice onto Columbia blood agar before testing.

### 4.3. Detection of mecA, icaA Gene, and SCCmec Typing

All *S. epidermidis* isolates were further tested to determine *mecA*, *icaA*, and SCC*mec* types using polymerase chain reaction (PCR) assays described by Zhang *et al*. [31]. This SCC*mec* PCR typing assay contained 9 pairs of primers including the unique and specific primers for SCC*mec* types and subtypes I, II, III, IVa, IVb, IVc, IVd, and V, and the primers for the *mecA* gene. We used *mecA* gene detection as the gold standard for confirming MRSE. The following reference strains were used: NCTC for *SCCmec* type I; N315 for *SCCmec* type II; 85/2080 for *SCCmec* type III; JCSC4744 for SCC*mec* type IV; and HS663 for SCC*mec* type V. The results were reported as type I-V, and those isolates that were not type I-V were deemed non-typeable (NT).

### 4.4. Antimicrobial Susceptibility Testing

Minimal inhibitory concentrations (MICs) of 18 antibiotics (penicillin, ampicillin, oxacillin, gentamicin, rifampicin, ciprofloxacin, gatifloxacin, levofloxacin, moxifloxacin, sulfamethoxazole, clindamycin, azithromycin, erythromycin, linezolid, vancomycin, chloramphenicol, quinupristin–dalfopristin, and tetracycline) were determined for MRSE isolates using the broth microdilution method as recommended by the Clinical and Laboratory Standards Institute (CLSI, Wayne, MI, USA) guidelines (M100-S25). Isolates were classified as multi-drug resistant (MDR) if they were resistant to ≥3 non-β-lactam antibiotic classes [32].

### 4.5. PFGE Typing

The *Sma*I DNA restriction fragments were separated by PFGE according to McDougal *et al.* [33]. Restriction fragments were separated using a Bio-Rad CHEF Mapper apparatus (Bio-Rad Laboratories Inc., Hercules, CL, USA). PFGE profiles obtained were analyzed with BioNumerics software (Applied Maths Inc., Austin, TX, USA). Clustering was performed by using the Dice similarity coefficient and the unweighted pair group method with arithmetic means (UPGMA), with optimization and position tolerance settings of 0.8 and 1.3, respectively. According to the criteria established by Miragaia *et al*. [15], a cutoff similarity value of 79% was used to establish PFGE types. The types obtained by the PFGE method are represented by numbers.

### 4.6. Quality Control

Standard aseptic sample collection and processing measures were strictly adhered to. For phenotypic identification of *S. epidermidis*, *S. epidermidis* ATCC 12228, and *S. aureus* ATCC 25923 were used as control strains, respectively. For genotyping, *S. aureus* ATCC 25923 (*mecA*-negative) and ATCC 43300 (*mecA*-positive) were used. For *icaA* gene PCR amplifying, *S. epidermidis* ATCC 12228 and ATCC 35984 were used as negative and positive controls respectively.

## 5. Conclusions

The results indicated that methicillin-resistant strains are frequently present among *S. epidermidis* on the abdominal skin of females before laparotomy. The MRSE isolates had an unfavorable antimicrobial susceptibility patterns and high genetic diversity, moreover resistance profiles seem to have no association with the SCC*mec* types or PFGE types for most common antibiotics.

## Figures and Tables

**Table 1:** Resistance profiles of 54 methicillin-resistant *S. epidermidis* (MRSE) isolates and 103 methicillin-susceptible *S. epidermidis* (MSSE) isolates.

Antibiotics	MSSE (*n* = 103)			MRSE (*n* = 54)			MIC Range
	%R(n)	MIC50	MIC90	%R(n)	MIC50	MIC90	
Penicillin G	85.4(88)	16	16	96.3(52)	16	16	0.03–16
Ampicillin	84.5(87)	16	16	96.3(52)	16	16	0.25–16
Oxacillin	0(0)	0.25	0.25	90.7(49)	4	4	0.25–4
Gentamicin	4.9(5)	4	4	14.8(8)	4	16	4–16
Rifampin	0(0)	1	1	0(0)	1	1	1–1
Ciprofloxacin	3.9(4)	1	1	29.6(16)	1	4	1–4
Gatifloxacin	0(0)	2	2	0(0)	2	2	2–2
Levofloxacin	2.9(3)	2	2	31.5(17)	2	8	2–8
Moxifloxacin	0(0)	2	2	0(0)	2	2	2–2
Trimethoprim/Sulfamethoxazole	25.2(26)	2	4	59.3(32)	4	4	2–4
Clindamycin	12.6(13)	0.5	2	14.8(8)	0.5	4	0.5–4
Azithromycin	38.8(40)	2	8	64.8(35)	8	8	2–8
Erythromycin	47.6(49)	0.5	8	68.5(37)	8	8	0.5–8
Linezolid	0(0)	2	2	0(0)	2	2	2–4
Vancomycin	0(0)	2	2	0(0)	2	4	2–16
Chloramphenicol	7.8(8)	8	8	11.1(6)	8	32	8–32
Quinupristin/Dalfopristin	0(0)	1	1	0(0)	1	1	1–1
Tetracycline	7.8(8)	4	4	9.3(5)	4	4	4–16

**Table 2:** Resistance profiles of 54 MRSE isolates with different staphylococcal cassette chromosome *mec* (SCC*mec*) types.

Antibiotics	Resistant Isolates of SCC*mec* Type [% (n)]						
	I (*n* = 1)	II + IVb (*n* = 1)	III (*n* = 4)	Iva (*n* = 28)	IVd (*n* = 3)	V (*n* = 3)	NT (*n* = 14)
Penicillin G	100(1)	100(1)	100(4)	96.4(27)	66.7(2)	100(3)	100(14)
Ampicillin	100(1)	100(1)	100(4)	96.4(27)	66.7(2)	100(3)	100(14)
Oxacillin	100(1)	100(1)	75(3)	89.3(25)	66.7(2)	100(3)	100(14)
Gentamicin	100(1)	0(0)	0(0)	10.7(3)	33.3(1)	0(0)	21.4(3)
Rifampin	0(0)	0(0)	0(0)	0(0)	0(0)	0(0)	0(0)
Ciprofloxacin	0(0)	100(1)	50(2)	21.4(6)	0(0)	66.7(2)	35.7(5)
Gatifloxacin	0(0)	0(0)	0(0)	0(0)	0(0)	0(0)	0(0)
Levofloxacin	0(0)	100(1)	50.0(2)	25.0(7)	0(0)	66.7(2)	35.7(5)
Moxifloxacin	0(0)	0(0)	0(0)	0(0)	0(0)	0(0)	0(0)
Trimethoprim/Sulfamethoxazole	0(0)	100(1)	25(1)	67.9(19)	66.7(2)	100(3)	42.9(6)
Clindamycin	0(0)	0(0)	25(1)	7.1(2)	0(0)	0(0)	35.7(5)
Azithromycin	100(1)	100(1)	25(1)	60.7(17)	66.7(2)	66.7(2)	78.6(11)
Erythromycin	100(1)	100(1)	25(1)	67.9(19)	66.7(2)	66.7(2)	78.6(11)
Linezolid	0(0)	0(0)	0(0)	0(0)	0(0)	0(0)	0(0)
Vancomycin	0(0)	0(0)	0(0)	0(0)	0(0)	0(0)	0(0)
Chloramphenicol	0(0)	0(0)	0(0)	7.1(2)	0(0)	0(0)	28.6(4)
Quinupristin/Dalfopristin	0(0)	0(0)	0(0)	0(0)	0(0)	0(0)	0(0)
Tetracycline	0(0)	100(1)	25(1)	7.1(2)	0(0)	0(0)	7.1(1)

**Table 3:** Resistance patterns of MRSE isolates.

Resistance Patterns	No. of Isol.	% Isol.	SCC*mec* Type (n)	PFGE Type (n)
ERY	2	3.7	IVa(2)	9(2)
SXT	1	1.9	IVa(1)	17(1)
OXA	4	7.4	III(1), IVa(1), IVd(1), NT(1)	1(4)
SXT + ERY	1	1.9	IVd(1)	1(1)
SXT + CIP	1	1.9	III(1)	25(1)
OXA + TCY	1	1.9	III(1)	28(1)
OXA + CHL	1	1.9	NT(1)	13(1)
OXA + ERY	3	5.6	IVa(1), NT(2)	16(1), 19(2)
OXA + CLI	2	3.7	IVa(2)	1(1), 12(1)
OXA + SXT	3	5.6	IVa(3)	20(1), 23(1), 24(1)
OXA + GEN	1	1.9	IVa(1)	12(1)
OXA + CLI + ERY	1	1.9	NT(1)	1(1)
OXA + SXT + CHL	1	1.9	IVa(1)	17(1)
OXA + SXT + ERY	6	11.1	IVa(5), V(1)	7(1),9(1),15(1), 7(1), 18(1), 19(1)
OXA + CIP + ERY	1	1.9	IVa(1)	22(1)
OXA + CIP + SXT	1	1.9	V(1)	1(1)
OXA + GEN + ERY	1	1.9	I(1)	12(1)
OXA + CLI + ERY + CHL	3	5.6	NT(3)	1(3)
OXA + SXT + ERY + TCY	2	3.7	IVa(2)	11(1), 17(1)
OXA + SXT + ERY + CHL	1	1.9	IVa(1)	15(1)
OXA +SXT + CLI + ERY	2	3.7	IVa(1), NT(1)	15(1), 29(1)
OXA + CIP + CLI + ERY	2	3.7	IVa(1), III(1)	2(1), 10(1)
OXA + CIP + SXT + ERY	5	9.3	IVa(2),V(1), NT(2)	1(2),8(1),14(1), 26(1)
OXA + GEN + SXT + ERY	1	1.9	IVd(1)	5(1)
OXA + GEN + CIP + SXT	1	1.9	NT(1)	3(1)
OXA + CIP + SXT + ERY + TCY	1	1.9	IVb/II(1)	11(1)
OXA + CIP + SXT + CLI + ERY	1	1.9	IVa(1)	21(1)
OXA + GEN + CIP + SXT + ERY	2	3.7	IVa(2)	6(1), 27(1)
OXA + GEN + CIP + SXT + ERY + CHL	1	1.9	NT(1)	3(1)
OXA + GEN + CIP + SXT + ERY + CHL + TCY	1	1.9	NT(1)	4(1)

ERY: erythromycin; SXT: sulfamethoxazole; OXA: oxacillin; CIP: ciprofloxacin; TCY: tetracycline; CHL: chloramphenicol; CLI: clindamycin; GEN: gentamicin.

## Abbreviations

MRSE	methicillin-resistant *Staphylococcus epidermidis*
MSSE	methicillin-susceptible *Staphylococcus epidermidis*
SCC*mec*	staphylococcal cassette chromosome *mec*
PFGE	pulsed field gel electrophoresis
NT	non-typeable
SSIs	surgical site infections
MICs	minimal inhibitory concentrations
